# Characterization of somatic mutations in sporadic uveal melanoma and uveal melanoma in patients with germline *BAP1* pathogenic variants

**DOI:** 10.1371/journal.pone.0306386

**Published:** 2024-10-08

**Authors:** Karin A. W. Wadt, Katja Harbst, Mette M. B. Sjøl, Frida Rosengren, Christina Westmose Yde, Kristoffer Staal Rohrberg, Marlene Richter Jensen, Steffen Heegaard, Jens Folke Kiilgaard, Anne-Marie Gerdes, Nicholas Hayward, Göran B. Jönsson

**Affiliations:** 1 Department of Clinical Genetics, Copenhagen University Hospital, Rigshospitalet, Copenhagen, Denmark; 2 Department of Cellular and Molecular Medicine, University of Copenhagen, Copenhagen, Denmark; 3 Division of Oncology and Pathology, Department of Clinical Sciences, Lund University Cancer Center, Lund University, Lund, Sweden; 4 Department of Ophthalmology, Copenhagen University Hospital, Rigshospitalet, Copenhagen, Denmark; 5 Genomic Medicine, Copenhagen University Hospital, Rigshospitalet, Copenhagen, Denmark; 6 Department of Oncology, Copenhagen University Hospital, Rigshospitalet, Copenhagen, Denmark; 7 Department of Clinical Medicine, University of Copenhagen, Copenhagen, Denmark; 8 Department of Pathology, Copenhagen University Hospital, Rigshospitalet, Copenhagen, Denmark; 9 QIMR Berghofer Medical Research Institute, Herston, QLD, Australia; Rutgers University, UNITED STATES OF AMERICA

## Abstract

Genetic analyses were conducted on tumor samples from 88 patients with uveal melanoma (UM), 6 of whom carry pathogenic germline variants in *BAP1*. We assessed the frequency, pattern, and prognostic significance of somatic aberrations, and investigated differences between germline *BAP1* variant carriers compared to sporadic cases. The frequency of the main oncogenic driver mutations was not significantly different between these groups. Patients with germline *BAP1* variants did not have significantly different overall survival compared to the wildtype or somatic *BAP1* mutation groups. Patients with a somatic *BAP1* mutation (n = 24) had a significantly worse prognosis compared to wildtype (n = 58). All patients with stage III tumors and a somatic *BAP1* mutation (n = 7) developed metastasis, however four of 28 stage I-II tumors without metastasis had somatic *BAP1* mutations, with observation time >5 years. The tumor from one germline *BAP1* carrier (stage IIIC) with a somatic *EIF1AX* splice variant, has not developed metastasis within a 22-year observation time.

## Introduction

Uveal melanoma (UM) is the most frequent adult primary tumor of the eye, with an incidence of 2–8 per million in Europe [1]. UM has a greater incidence in countries with higher latitude in Europe and North America, and meta-analysis has shown increased risk with light skin color and blue or green eye color [2]. UM occurs in the choroid, ciliary body or iris, and only the latter is potentially exposed to UV-radiation. However, the molecular profiles of UM and cutaneous melanoma (CM) are different, with a distinctive mutation spectrum in UM and no UV-mutation signatures described in choroidal or ciliary body UM [3]. The vast majority of UM have a somatic gain-of-function Gα_q_ pathway mutation in either *GNAQ*, *GNA11*, *CYSLTR2* or *PLCB4* [4–6]. Additionally, somatic chromosomal aberrations including monosomy 3, 8q gain, and loss of 1p, are frequent and associated with poor prognosis [7, 8]. Recently, extensive somatic mutation profiling has divided UM into 4 classes [3], with the most significant factor associated with adverse prognosis and high metastatic risk being *BAP1* mutation/loss [9–13].

Recent advances in treatment of CM have shown enormous improvement in patient outcome [14], however, this success has not been translated to UM [15]. Around 50% of UM metastasize, preferentially to the liver [16] and at present no curable treatment has been identified. On average, patients survive 3.9 months after diagnosis of liver metastasis [17]. A recent retrospective study of UM patients with metastases treated with immune checkpoint inhibitors showed that germline and/or somatic *MBD4* mutations are highly predictive of response [18]. Such mutations are rare but illustrates the importance of further molecular subdivision of UM [18, 19].

Here, we assessed the frequency, pattern, and prognostic significance of somatic mutations in a Danish cohort of UM patients, and investigated differences between germline *BAP1* variant carriers compared to sporadic cases.

## Results

Molecular data were obtained from 80 primary UM tumors and 8 metastases. Clinical data for the 88 patients is presented in S1 Table. Of the 82 patients with sporadic UM, 33 (40%) did not develop metastasis (minimum follow-up time of 5 years, range 5–36 years), while 49 (60%) developed metastasis. Five of the six patients with a germline *BAP1* pathogenic variant (PV) developed metastases. None of the patients with a germline *BAP1* PV had received presymptomatic testing or surveillance, as their variants were identified following their UM diagnosis. The tumor from the germline *BAP1* PV carrier with a stage IIIC UM, and no metastasis (observation time 22 years) had an *EIF1AX* splice mutation (c.429+1G>A). This tumor has been reported to have loss of the wildtype *BAP1* allele [20]. Of the 6 *BAP1* germline PV carriers, two tumors carried a less frequently reported *GNA11* mutation (R183C), and one tumor the less frequent variant p.Q209H in GNAQ, whereas the others had the frequent *GNA11/GNAQ* hotspot mutations at codon 209. The frequency of germline or somatic *BAP1* mutations is significantly higher in the metastasis group (46%) compared to the non-metastasis group (15%) (p = 0.0005).

**Fig 1:**
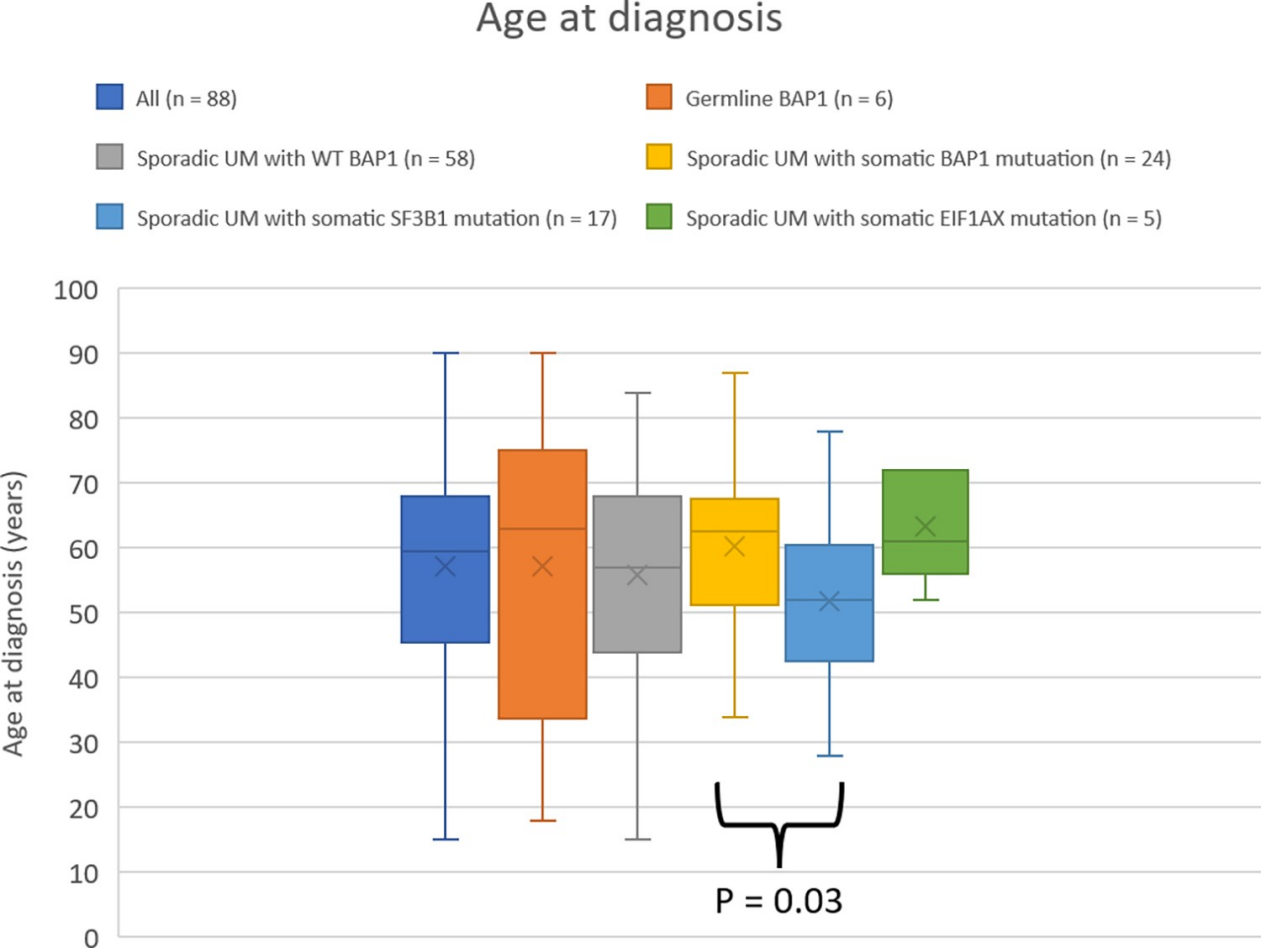
Age at diagnosis of UM in the total cohort and different subgroups. Individuals with somatic *BAP1* mutation were significantly older at diagnosis than individuals with *SF3B1* mutation (p = 0.03).

Of sporadic UM patients, 24 (29%) carried a *BAP1* somatic mutation classified as pathogenic or likely pathogenic. One patient carried the *BAP1* variant c.2057-4G>T previously reported likely benign [21–23], resulting in this patient being categorized in the wild-type *BAP1* group. In sporadic UM patients with tumors that had somatic *BAP1* pathogenic mutations, age at diagnosis (average 60 years), was not significantly older compared to the total cohort (average 57 years) or to patients with wild-type *BAP1* (average 56 years), but significantly older than cases with *SF3B1* mutations (average 52 years) (*p* = 0.03) (Fig 1).

**Fig 2:**
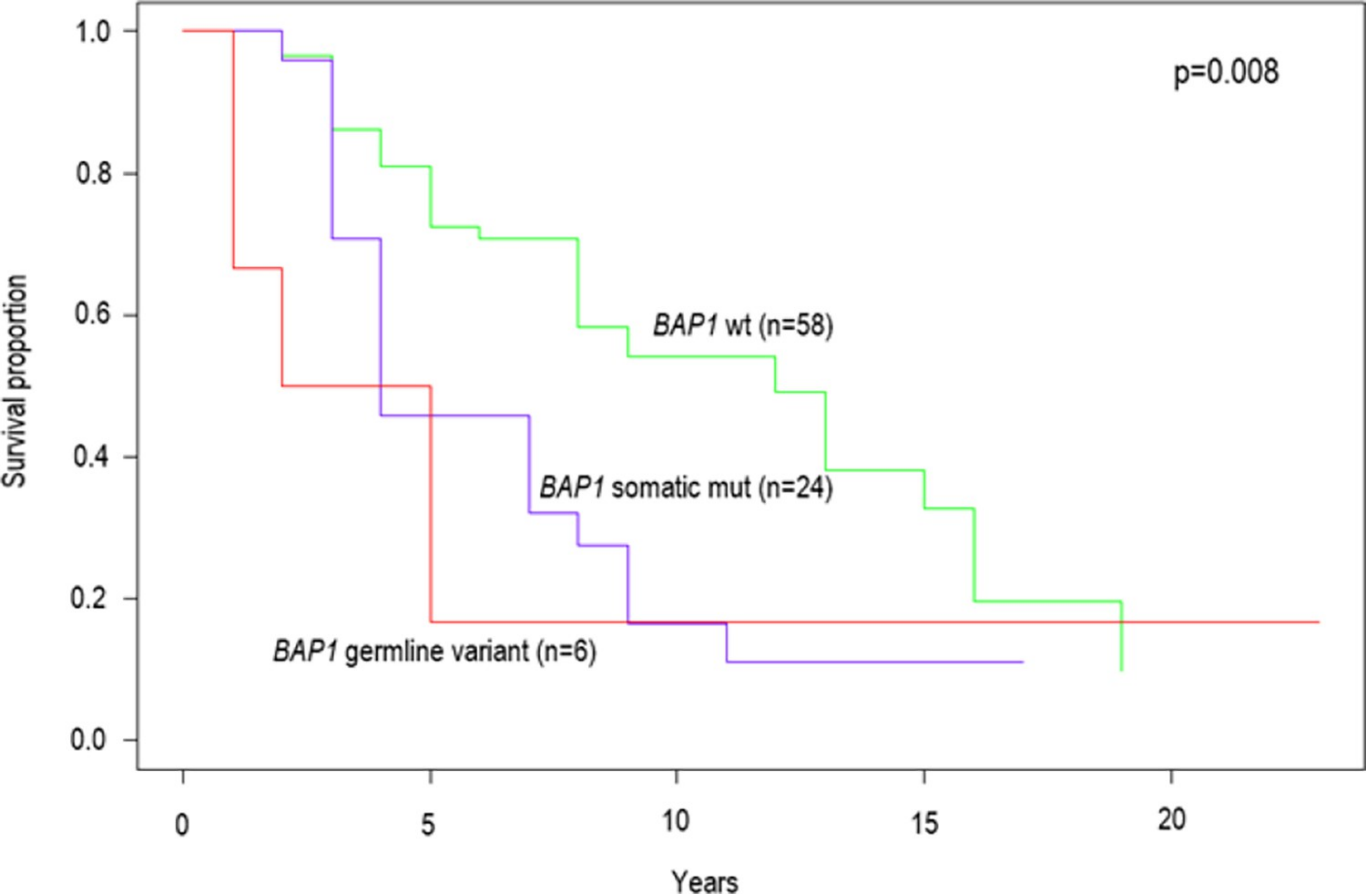
Kaplan-Meier curve showing the association of *BAP1* status with overall survival (OS). X-axis represents years after UM diagnosis, Y-axis represents the survival fraction. P-values were derived from Cox log-rank test (p = 0.008) and Cox regression between the three groups, showing significantly worse OS of somatic *BAP1* mutation carriers than wildtype *BAP1* carriers (p = 0.005).

Sporadic UM patients with a *BAP1*-mutant tumor, had significantly worse overall survival (OS) (p = 0.005, Cox log-rank test) compared to those with wildtype *BAP1* (Fig 2). Patients with germline *BAP1* PVs (n = 6) did not have significantly different OS compared to the wildtype *BAP1* (p = 0.09) or the somatic *BAP1* mutation group (p = 0.99) (Fig 2). No significant differences in OS were seen in relation to *GNAQ*, *GNA11* or *SF3B1* mutations (p = 0.1, p = 0.9, p = 0.9, respectively).

**Fig 3:**
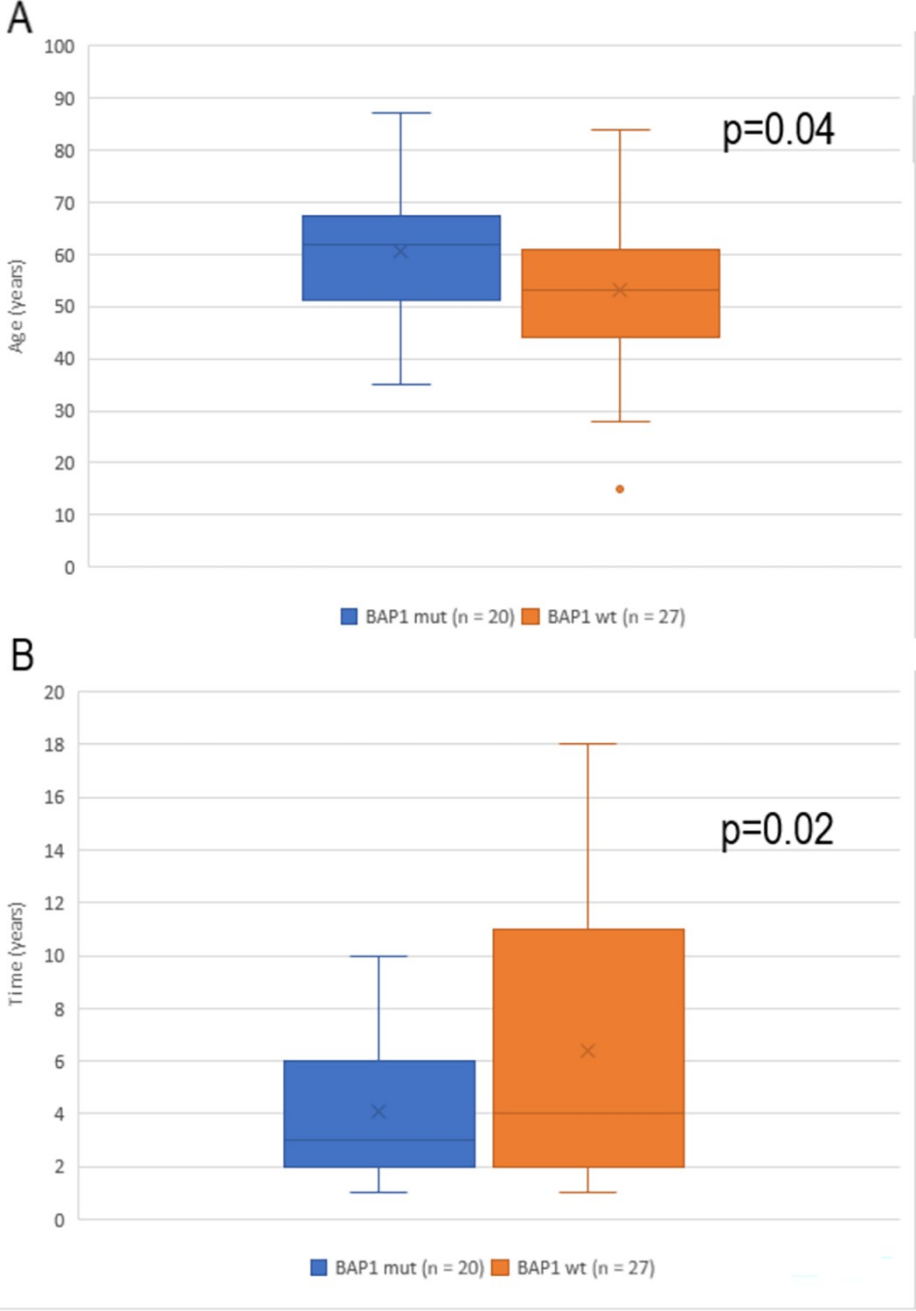
Comparison of sporadic UM patients who developed metastasis, with and without somatic *BAP1* mutation. Patients with somatic *BAP1* mutation had significantly older age at diagnosis (p = 0.04) (A) and shorter time to death from UM diagnosis (p = 0.02); (B) despite comparable AJCC clinical stage in the two groups.

Sporadic UM patients who developed metastasis (n = 47) were significantly older at time of diagnosis if their tumors had a somatic *BAP1* mutation (n = 20, average 61 years) compared to wildtype *BAP1* (n = 27, average 53 years) (p = 0.04) and shorter time to death from diagnosis, which was on average 4.1 years in the *BAP1* somatic mutation group, compared to 6.4 years in tumors with no somatic *BAP1* mutation (p = 0.02) (Fig 3). This result was despite quite comparable AJCC clinical staging of the tumors, with a median stage of IIIA in the *BAP1* wildtype group, and IIB in the *BAP1* somatic mutation group (data not available for 9 patients: 5 in the *BAP1* somatic mutation group and 4 in non-*BAP1* somatic mutation group). No mutations in *BAP1* were found in stage III tumors with no metastasis, whereas 4 of 28 stage I-II tumors without metastasis had *BAP1* mutations. These patients have been observed for 5 †, 7 †, 10, and 16 years.

**Fig 4:**
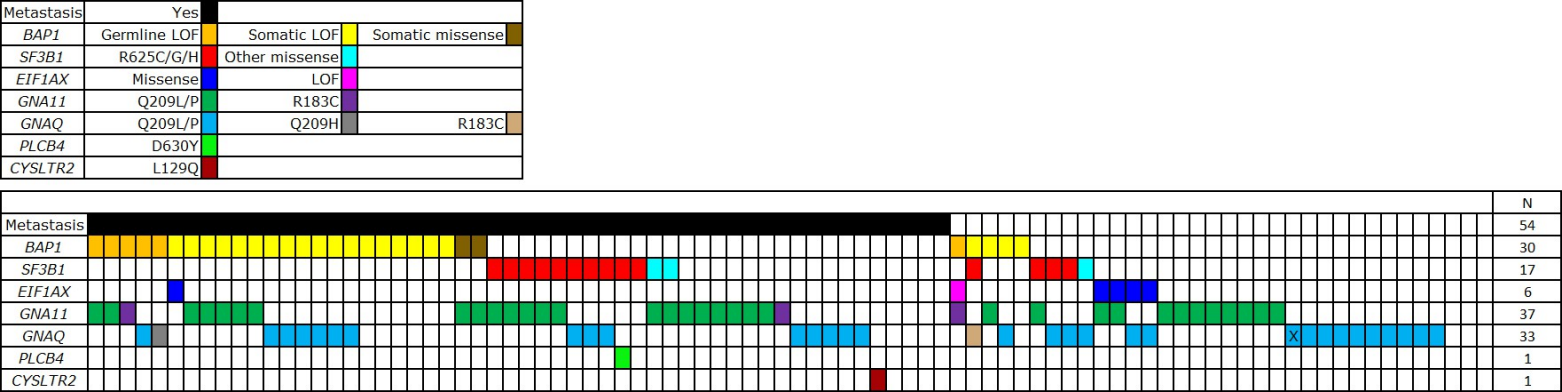
Mutations identified in the study. Individual genes are represented as rows. Individual patients are represented as columns. The mutation type is shown by the color. One patient with family history of UM is marked by “X”.

Fig 4 shows mutations in tumors from each of the 88 patients, clustered by those with or without metastasis. Somatic mutations in the Gα_q_ signaling pathway genes (i.e. *GNAQ*, *GNA11*, *PLCB4*, *CYSLTR2)*, as well as *SF3B1* and *EIF1AX*, were mutually exclusive. The frequency of *GNA11* and *GNAQ* mutations was not significantly different between tumors with and without *BAP1* mutation (p = 0.14, chi^2^ test) or between tumors with germline or somatic *BAP1* mutations (p = 0.08, chi^2^ test).

Rare variants and mutations identified in the study that are not normally seen in UM include a germline *RB1* VUS (variant of uncertain significance) (p.A488T), a *KIT* p.V422M VUS, previously described germline, in this dataset unknown if germline or somatic, a *NRAS* p.*E63V* VUS, not previously reported germline and unknown somatic or germline in this dataset. Further, a tumor with a *CDKN2A* mutation *c*.*233_234delTC*, also harbored a truncating *BAP1* and a *GNA11* driver mutation. These rare variants were each identified in one patient (S1 Table).

## Discussion

In this study we assessed the frequency, pattern, and prognostic significance of somatic aberrations in 82 sporadic UM and 6 UM from germline pathogenic *BAP1* variant carriers. *EIF1AX* is mutated in approximately 20% of UM [24] and such UM have been shown to rarely metastasize and have better prognosis compared to UM without *EIF1AX* mutation [13, 24]. One germline *BAP1* pathogenic variant carrier has not developed metastasis after 22 years of observation and we speculate this is because the tumor had a somatic splice mutation in *EIF1AX*. To our knowledge, no other studies have evaluated somatic *EIF1AX* mutations in germline *BAP1* PV carriers. In the other five germline *BAP1* variant carriers we found only *GNAQ* and *GNA11* somatic mutations.

As expected from previous studies [25, 26], we found a significantly worse prognosis associated with somatic *BAP1* mutations compared to wildtype (Fig 2). The Kaplan-Meier curve as well suggests worse OS of germline *BAP1* variant carriers compared to wildtype but analysis is limited by small sample size (n = 6). A study by Ewens et al., 2018 [25] directly compared the risk of UM metastasis between tumors with germline *BAP1* variants, somatic *BAP1* mutations and wild type *BAP1* tumors. They found tumors with somatic *BAP1* mutations metastasized significantly more often compared to both germline and wildtype. Patients with germline *BAP1* variants did not show significantly different prognosis than patients with wildtype *BAP1* tumors, which however could be due to low numbers, i.e. of the six *BAP1* germline variant carriers in this cohort, five died within 0–4 years following UM diagnosis and one was a long-term survivor (22 years). No significant alterations in survival were seen in relation to *GNAQ*, *GNA11* or *SF3B1* mutations (p = 0.1, p = 0.9, p = 0.9, respectively), in keeping with previous findings [9, 10, 13].

We found a significantly older age at diagnosis in patients with tumors that had *BAP1* somatic mutations (n = 24) compared to patients with tumors with *SF3B1* mutations (n = 17, p = 0.03, Fig 1). It has been reported by Ewens et al. 2018 [25], as well as others [26, 27], that patients with UM somatic *BAP1* mutations have a significantly older age at diagnosis compared to wildtype, in agreement with our study, although not significantly different. As pointed out by others [9], performing *BAP1* exonic sequencing, will not identify all *BAP1* mutations, but unfortunately due to lack of tumor tissue we were not able to perform BAP1 IHC or chromosome copy number analysis on the tumors, which would have made the analysis more complete. We did however find a significantly shorter time to death in patients who developed metastasis and carry a *BAP1* somatic mutation compared to patients who developed metastasis without a somatic *BAP1* mutation.

Finally, we identified somatic *BAP1* mutations in stage I-II tumors from patients who have not developed metastasis, whereas we do not find any *BAP1* somatic mutations in stage III-IV tumors with no metastasis, speculatively indicating perhaps the existence of a threshold: i.e., in stage I-II tumors metastasis may develop if a somatic *BAP1* mutation is found, but in stage III-IV metastasis is inevitable if somatic *BAP1* mutations are present.

## Materials and methods

### Patient

Tumors from 100 persons with primary uveal melanoma treated with enucleation from 1986 to 2011 were selected and permissions granted from the Ethics committee of the Capital region of Copenhagen. H-15005619. In the retrospective cohort clinical data from participants was obtained from 1 September 2015 to 31 October 2020. During the study period the authors could identify individual participants. The Ethics committee waived the requirement for informed consent.

The UM included those from four known germline *BAP1* mutation carriers, 48 samples of metastatic UM and 48 UM with no metastasis and at least 5 years follow-up. Of the 100 samples, molecular genetic results were obtained from 81 samples. During the duration of the project four patients developed metastasis and their group status was changed. In one patient a *KRAS* p.G12V mutation was found, and pathology reexamination was performed and a uveal metastasis from an undetected cutaneous melanoma could not be excluded, thus the patient was omitted from the dataset. In total, molecular data were obtained from primary tumors of four UM cases with germline *BAP1* mutation, 41 samples of UM (metastasized) and 35 samples of UM with no metastasis.

In addition to the 80 patients, sequencing data from metastases from 8 patients (1 germline *BAP1* PV) were included from a clinical pipeline. Materials and methods performed included whole-exome sequencing on germline DNA from blood samples and DNA from matched tumor biopsies, and have been described elsewhere [28] and Regulatory approvals from the Regional Ethics Committee and the Danish Data Protection Agency were obtained (Danish Ethical Committee, file number: 1300530). All patients provided signed informed written consent. Information on AJCC clinical staging of the tumors was not available for these 8 patients.

Clinical data regarding the 88 patients are presented in S1 Table.

### Samples

FFPE tissue blocks from 100 UM where cut in 8 μm sections; 5 slides from each tumor unstained on glass and one slide from each tumor stained with H&E using a standard protocol. Under a microscope the samples were dissected and normal tissue separated from tumor tissue.

### DNA extraction and sequencing

DNA and RNA were extracted from the tumor and normal tissue using Allprep DNA/RNA FFPE extraction kit (Qiagen, catalog number 80234). DNA was submitted to quality control using FFPE DNA Library Prep QC Kit (Illumina, catalog number FC-121-9999) and only tumor samples with delta CT of 10 or less were selected for sequencing library preparation. Libraries were prepared from tumor DNA using TruSeq custom amplicon low input kit (Illumina) and sequenced on a NextSeq500 (Illumina). The custom gene panel comprised the following genes: *GNA11*, *GNAQ*, *BAP1*, *SF3B1*, *EIF1AX*, *EGFR*, *NRAS*, *ARID2*, *KIT*, *FBXW7*, *NF1*, *CDK4*, *CDKN2A*, *TP53*, *RAC1*, *RB1*, *KRAS*, *STK11*, *CTNNB1*.

Sanger sequencing of *PLCB4* (D630Y) and *CYSLTR2* (L129Q), in tumors without a *GNA11*, *GNAQ*, or *BAP1* variant (n = 8).

### Statistical analyses

Continuous variables were compared using the *t*-test. The overall survival (OS) curves were generated by the Kaplan-Meier method, and OS between groups compared using Cox regression. A p < 0.05 was deemed statistically significant in all calculations. Kaplan-Meier, log-rank test and Cox regression were carried out with the R software version 4.3.

## Supporting information

S1 Table Clinical data and genetic results from the 88 patients. Germline data shown with color brown.
(XLSX)

## References

[1] Virgili G, Gatta G, Ciccolallo L, Capocaccia R, Biggeri A, Crocetti E, et al. Incidence of Uveal Melanoma in Europe. Ophthalmology. 2007;114:2309–2315. doi: 10.1016/j.ophtha.2007.01.032 17498805

[2] Weis E, Shah CP, Lajous M, Shields JA, Shields CL. The Association Between Host Susceptibility Factors and Uveal Melanoma: A Meta-analysis. Arch Ophthalmol. 2006;124:54–60. doi: 10.1001/archopht.124.1.54 16401785

[3] Robertson AG, Shih J, Yau C, Gibb EA, Oba J, Mungall KL, et al. Integrative Analysis Identifies Four Molecular and Clinical Subsets in Uveal Melanoma. Cancer Cell. 2017;32:204–20. doi: 10.1016/j.ccell.2017.07.003 28810145 PMC5619925

[4] Johansson P, Aoude LG, Wadt K, Glasson WJ, Warrier SK, Hewitt AW, et al. Deep sequencing of uveal melanoma identifies a recurrent mutation in PLCB4. Oncotarget. 2016;7:4624–31. doi: 10.18632/oncotarget.6614 26683228 PMC4826231

[5] Moore AR, Ceraudo E, Sher JJ, Guan Y, Shoushtari AN, Chang MT, et al. Recurrent activating mutations of G-protein-coupled receptor CYSLTR2 in uveal melanoma. Nat Genet [Internet]. 2016;48(6):675–80. Available from: doi: 10.1038/ng.3549 27089179 PMC5032652

[6] Johansson PA, Brooks K, Newell F, Palmer JM, Wilmott JS, Pritchard AL, et al. Whole genome landscapes of uveal melanoma show an ultraviolet radiation signature in iris tumours. Nat Commun [Internet]. 2020;11(1):1–8. Available from: 10.1038/s41467-020-16276-8 32415113 PMC7229209

[7] Horsthemke B, Prescher G, Bornfeld N, Becher R. Loss of chromosome 3 alleles and multiplication of chromosome 8 alleles in uveal melanoma. Genes Chromosom Cancer. 1992;4(3):217–21. doi: 10.1002/gcc.2870040305 1382562

[8] Dogrusöz M, Bagger M, Van Duinen SG, Kroes WG, Ruivenkamp CAL, Böhringer S, et al. The prognostic value of AJCC staging in uveal melanoma is enhanced by adding chromosome 3 and 8q status. Investig Ophthalmol Vis Sci. 2017;58(2):833–42. doi: 10.1167/iovs.16-20212 28159971

[9] Field MG, Durante MA, Anbunathan H, Cai LZ, Decatur CL, Bowcock AM, et al. Punctuated evolution of canonical genomic aberrations in uveal melanoma. Nat Commun [Internet]. 2018;9(116). Available from: doi: 10.1038/s41467-017-02428-w 29317634 PMC5760704

[10] Staby KM, Gravdal K, Mørk SJ, Heegaard S, Vintermyr OK, Krohn J. Prognostic impact of chromosomal aberrations and GNAQ, GNA11 and BAP1 mutations in uveal melanoma. Acta Ophthalmol. 2018;96:31–8. doi: 10.1111/aos.13452 28444874

[11] Silva-Rodríguez P, Fernández-Díaz D, Bande M, Pardo M, Loidi L, Blanco-Teijeiro MJ. GNAQ and GNA11 Genes: A Comprehensive Review on Oncogenesis, Prognosis and Therapeutic Opportunities in Uveal Melanoma. Cancers (Basel). 2022;14(13). 10.3390/cancers14133066 PMC9264989 35804836

[12] Griewank KG, Van De Nes J, Schilling B, Moll I, Sucker A, Kakavand H, et al. Genetic and clinico-pathologic analysis of metastatic uveal melanoma. Mod Pathol [Internet]. 2014;27(2):175–83. Available from: doi: 10.1038/modpathol.2013.138 23887304

[13] Martin M, Maßhöfer L, Temming P, Rahmann S, Metz C, Bornfeld N, et al. Exome sequencing identifies recurrent somatic mutations in EIF1AX and SF3B1 in uveal melanoma with disomy 3. Nat Genet. 2013;45(8):933–6. doi: 10.1038/ng.2674 23793026 PMC4307600

[14] Pasquali S, Hadjinicolaou A V., Chiarion Sileni V, Rossi CR, Mocellin S. Systemic treatments for metastatic cutaneous melanoma. Cochrane Database Syst Rev. 2018;2(2). doi: 10.1002/14651858.CD011123.pub2 29405038 PMC6491081

[15] Johnson DB, Daniels AB. Continued poor survival in metastatic uveal melanoma implications for molecular prognostication, surveillance imaging, adjuvant therapy, and clinical trials. JAMA Ophthalmol. 2018;136(9):986–8. doi: 10.1001/jamaophthalmol.2018.1813 29955760

[16] Singh AD, Bergman L, Seregard S. Uveal melanoma: epidemiologic aspects. Ophthalmol Clin North Am. 2005;18(1):75–84. doi: 10.1016/j.ohc.2004.07.002 15763193

[17] Lane AM, Kim IK, Gragoudas ES. Survival rates in patients after treatment for metastasis from uveal melanoma. JAMA Ophthalmol. 2018;136(9):981–6. doi: 10.1001/jamaophthalmol.2018.2466 29955797 PMC6142974

[18] Saint-Ghislain M, Derrien AC, Geoffrois L, Gastaud L, Lesimple T, Negrier S, et al. MBD4 deficiency is predictive of response to immune checkpoint inhibitors in metastatic uveal melanoma patients. Eur J Cancer. 2022;173:105–12. doi: 10.1016/j.ejca.2022.06.033 35863105

[19] Nathan P, Hassel JC, Rutkowski P, Baurain J-F, Butler MO, Schlaak M, et al. Overall Survival Benefit with Tebentafusp in Metastatic Uveal Melanoma. N Engl J Med. 2021;385(13):1196–206. doi: 10.1056/NEJMoa2103485 34551229

[20] Wadt K, Choi J, Chung JY, Kiilgaard J, Heegaard S, Drzewiecki KT, et al. A cryptic BAP1 splice mutation in a family with uveal and cutaneous melanoma, and paraganglioma. Pigment Cell Melanoma Res. 2012;25(6):815–8. doi: 10.1111/pcmr.12006 22889334 PMC7453745

[21] Boru G, Grosel TW, Pilarski R, Stautberg M, Massengill JB, Jeter J, et al. Germline large deletion of BAP1 and decreased expression in non-tumor choroid in uveal melanoma patients with high risk for inherited cancer. Genes Chromosom Cancer. 2019;58(9):650–6. doi: 10.1002/gcc.22752 30883995 PMC6612571

[22] Betti M, Casalone E, Ferrante D, Romanelli A, Grosso F, Guarrera S, et al. Inference on germline BAP1 mutations and asbestos exposure from the analysis of familial and sporadic mesothelioma in a high-risk area. Genes Chromosom Cancer. 2015;54(1):51–62. doi: 10.1002/gcc.22218 25231345

[23] Pilarski R, Cebulla CM, Massengill JB, Rai K, Rich T, Strong L, et al. Expanding the clinical phenotype of hereditary BAP1 cancer predisposition syndrome, reporting three new cases. Genes Chromosom Cancer. 2014;53(2):177–82. doi: 10.1002/gcc.22129 24243779 PMC4041196

[24] Smit KN, Jager MJ, de Klein A, Kiliҫ E. Uveal melanoma: Towards a molecular understanding. Prog Retin Eye Res. 2020;75(April 2019). doi: 10.1016/j.preteyeres.2019.100800 31563544

[25] Ewens KG, Lalonde E, Richards-Yutz J, Shields CL, Ganguly A. Comparison of Germline versus Somatic BAP1 Mutations for Risk of Metastasis in Uveal Melanoma. BMC Cancer. 2018;18(1):1–12. 30477459 10.1186/s12885-018-5079-x PMC6260582

[26] Yavuzyigitoglu S, Koopmans AE, Verdijk RM, Vaarwater J, Eussen B, Van Bodegom A, et al. Uveal Melanomas with SF3B1 Mutations: A Distinct Subclass Associated with Late-Onset Metastases. Ophthalmology [Internet]. 2016;123(5):1118–28. Available from: doi: 10.1016/j.ophtha.2016.01.023 26923342

[27] Kalirai H, Dodson A, Faqir S, Damato BE, Coupland SE. Lack of BAP1 protein expression in uveal melanoma is associated with increased metastatic risk and has utility in routine prognostic testing. Br J Cancer. 2014;111(7):1373–80. doi: 10.1038/bjc.2014.417 25058347 PMC4183849

[28] Bertelsen B, Tuxen IV, Yde CW, Gabrielaite M, Torp MH, Kinalis S, et al. High frequency of pathogenic germline variants within homologous recombination repair in patients with advanced cancer. npj Genomic Med [Internet]. 2019;4(1). Available from: doi: 10.1038/s41525-019-0087-6 31263571 PMC6588611

